# Temporal Dynamics of Reproduction in *Hemiramphus brasiliensis* (Osteichthyes: Hemiramphidae)

**DOI:** 10.1155/2014/837151

**Published:** 2014-11-16

**Authors:** Mônica Rocha de Oliveira, Sathyabama Chellappa

**Affiliations:** Departamento de Oceanografia e Limnologia, Centro de Biociências, Universidade Federal do Rio Grande do Norte, Via Costeira Senador Dinarte Medeiros Mariz, Mãe Luíza, s/n, 59.014-002 Natal, RN, Brazil

## Abstract

The reproductive aspects of *Hemiramphus brasiliensis* were analyzed with a view to verify the temporal dynamics of reproduction. This paper presents data on sex ratio, length at first sexual maturity, macroscopic and histological aspects of gonad development, gonadosomatic index (GSI), reproductive period, and fecundity of *H. brasiliensis*. The fishes were captured from the coastal waters of Rio Grande do Norte, northeastern Brazil. Females of this species predominated in the sampled population and were larger in size than the males. The length at the first sexual maturation of males was 20.8 cm and that of females was 21.5 cm. The macroscopic characteristics of the gonads indicated four maturation stages. Histological studies of gonads of *H. brasiliensis* showed six phases of oocyte development and four phases of spermatocyte development. The batch fecundity of this species was 1153 (±258.22) mature oocytes for 50 g body weight of female. The microscopic characteristics of gonad development indicate that *H. brasiliensis* is a multiple spawner, presenting a prolonged reproductive period during the whole year, with a peak in the month of April, and is considered as an opportunistic strategist.

## 1. Introduction

Reproductive strategies of fish are used to maximize production and ensure the survival of offspring to adulthood. Each strategy is expressed by various tactics, such as size at first maturation, fecundity, and spawning period, which are considered as important information for making rational measures to regulate fishing and conservation of fish stocks [1, 2]. Studies on the life history of the animals show that r-strategists are characterized by small body size with early first sexual maturity, whereas the K-strategists have long lifespan, large body size, and late sexual maturity [3–5]. Kawasaki [6, 7] suggested that the grouping of life history traits of marine fishes differed from the traditional r- and K-strategists developed for terrestrial animals [3, 5], As such, a third intermediary group was suggested for marine fish characterized by long lifespan with large body size and early sexual maturity [6, 7]. Clusters of life history strategies based on 16 characteristics using a large sample of 216 species of marine and freshwater fish from North America were developed [8]. Later, they made a selection of five life history traits for 82 species of freshwater and 65 marine fishes and suggested a model of three strategies: (1) opportunistic strategists with small body size, early first sexual maturity, and short-life span; (2) periodic or seasonal strategists of big body size with long life span and high to intermediate fecundity; and (3) equilibrium strategists of intermediate size in relatively stable environment [2].

The fishes popularly known as ballyhoo half beak belong to the family Hemiramphidae [9] and are found in shallow marine and estuarine waters in the Atlantic, Indian, and Pacific Oceans [10]. The following species have been registered in the coastal waters of Brazil:* Hemiramphus brasiliensis*,* Hemiramphus balao*,* Hyporhamphus roberti*,* Hyporhamphus unifasciatus*,* Euleptorhamphus velox*,* Hyporhamphus coroa,* and* Hyporhamphus salvatoris* [11]. Among these species,* H. brasiliensis* and* H. unifasciatus* are commercially important [12].* H. brasiliensis* (Linnaeus, 1758) is encountered in the entire Tropical Atlantic Ocean, occurring on both sides of the Atlantic Ocean, from New England to the Southeast of Brazil, preferring calm and warm waters near the coast [13, 14]. These fish are small in size with a maximum length of 30 cm [15] and are important for artisanal fisheries as bait and as food [12, 16, 17].

South Florida's lampara net fishery has been a small but valuable bait fishery targeting halfbeaks (Hemiramphidae) [18]. Two halfbeak species, ballyhoo,* H. brasiliensis,* and balao,* H. balao*, are harvested as bait in south Florida waters, and changes in fishing effort and regulations prompted an investigation of the overlap of halfbeak fishing grounds and spawning grounds. It was observed that both halfbeak species spawned throughout the fishing grounds of south Florida [14]. Reproductive biology of* H. brasiliensis* and* H. balao* relating to maturation, spawning frequency, and fecundity were reported from the coastal waters of southeastern Florida, USA [14]. Another study examined the development, reproduction, and symbiosis of an isopod parasite associated with its host* H. brasiliensis* [19]. Considering the importance of* H. brasiliensis*, this work presents data on temporal dynamics and reproductive strategy of this species occurring in the Northeastern coastal waters of Brazil.

## 2. Materials and Methods

### 2.1. Study Site and Sample Collection

The municipality of Caiçara do Norte is first among the top five areas of fish production in the state Rio Grande do Norte, Brazil. Artisanal fishing is the main economic activity of this municipality, producing a wide variety of commercially important marine fish, such as the flying fish,* Hirundichthys affinis*; dolphinfish,* Coryphaena hippurus;* yellowfin tuna,* Thunnus albacares;* black grouper,* Mycteroperca bonaci;* lane snapper,* Lutjanus synagris;* mutton snapper,* L. analis;* southern red snapper,* L. purpureus;* grey snapper,* L. griseus;* dog snapper,* L. jocu;* yellowtail snapper,* Ocyurus chrysurus*; thread herring,* Opisthonema oglinum*; white mullet,* Mugil curema*; and fantail mullet,* M. liza*. In addition, small inshore fish such as the ballyhoo halfbeak,* H. brasiliensis* is also captured. These fish are captured by local fishermen using different types of fishing crafts and nets [17].

*H. brasiliensis* samples were captured on a monthly basis from the inshore waters of Caiçara do Norte, northeastern Brazil, during the period of May 2011 to April 2012 (34°59′ to 37°14′ longitude W and 4°54′ to 6°34′ latitude S). The inshore waters have gradually sloping sandy banks, covered by macroalgal vegetation. Fish samples were captured with the help of local artisanal fishermen, who used small traditional fishing boats and the ballyhoo fishing nets. These nets are made of nylon, with 120 m in length, 2.5 m in height, a mesh size of 20 mm in the central part, and 40 mm in the extremities.* H. brasiliensis* samples were randomly collected from catch landed by fishermen. Other small fish species captured were discarded. During the months of September and December 2011 fish were not captured, due to the strong winds that prevailed which prevented the fishermen from fishing activities. Fish captured during the study period were numbered, weighed in total body weight (*W*_*t*_ to the nearest gram, g) and measured in total body length (*L*_*t*_ to the nearest centimeter, cm) for males and females (mean ± SD). Each fish was dissected and sex was identified based on the macroscopic characteristics of the gonads [20]. The gonad weight was measured (to the nearest 0.1 g). The *t*-test was utilized to check the difference between the total body length and weight of males and females [21].

### 2.2. Sex Ratio

The sex ratio was given as males : females (M : F), calculated using the formula: total number of males/total number of females. The chi-square (*χ* ^2^) was used to verify the existence of significant differences between the sex ratio of the study species and commonly expected 1 : 1 sex ratio [21].

### 2.3. Body Size at First Gonadal Maturity (*L*_50_)

Body size at first gonadal maturity (*L*_50_) where 50% of the individuals exhibited maturing gonads was estimated from the relative frequency distribution of adult males and females, using their standard length classes (mean ± SD) [22].

### 2.4. Macroscopic and Histological Descriptions of the Gonads

The location and general aspects of the gonads were observed and stage of reproductive maturity was determined using a macroscopic staging system. The features used for the macroscopic classification of gonads were based on the following external aspects: size, shape, color, presence of blood vessels, stiffness, and the space occupied in the coelomic body cavity [23].

In order to avoid possible variation in the developmental stage of oocytes, due to their position in the ovaries, histological examinations were carried out on sections from the anterior (cephalic), middle (central), and posterior (caudal) regions of 20 ovaries in different developmental stages [24]. These data were later compared in order to determine whether samples taken from midsection of the ovary of either lobe were representative of oocyte development.

Fragments of the ovaries and testicles were preserved in Bouin's solution for 12 to 24 hours, later embedded in paraffin, sectioned at 3–5 *μ*m thickness, and stained with Harris Hematoxylin and Eosin (HE) and periodic acid Schiff (PAS). Gonadal developmental stages were assessed microscopically with the help of light microscope (Taimin, model TM 800), coupled with a video camera (Kodo Digital). The terminology used for staging of oogenesis was based on existing information [25–28].

### 2.5. Fecundity

To determine the fecundity, ovaries were removed from 14 mature females varying in size from 22 to 24 cm. The gonad was weighed (to the nearest 0.1 g) and then preserved in Gilson solution for 24 hours for complete dissociation of oocytes, which were then washed and preserved in 70% ethyl alcohol. A 10% sample was removed for counting of whole oocytes in final maturation, using Bogorov plates, a stereo microscope, and an ocular micrometer, and the values were extrapolated to 100% [23]. Regression analysis was utilized to check the correlation between body weight of females and fecundity and weight of ovary and fecundity.

### 2.6. Estimation of the Gonadosomatic Index (GSI) and the Spawning Period

The gonadosomatic index (GSI) was calculated for maturing and mature female fish separately: GSI = weight of ovary (g)/body weight of fish (g) − weight of gonads (g) × 100 [29]. Reproductive period was determined by the temporal relative frequency distribution of the different stages of ovarian maturation [14, 30, 31].

## 3. Results and Discussion

**Figure 1:**
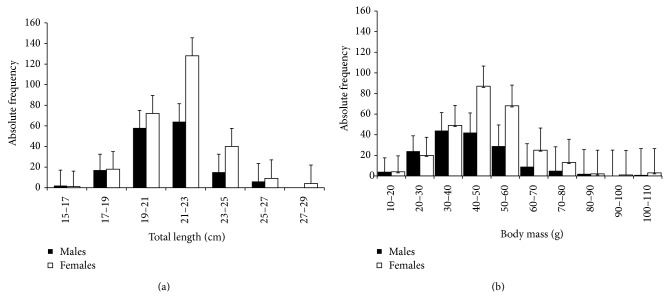
Mean monthly distribution according to class intervals of (a) total body length; (b) body mass of* H. brasiliensis* (*N* males = 160; *N* females = 272).

A total of 432 fishes were captured (160 males and 272 females). Figures 1(a) and 1(b) show the distribution of total length and total body weight of males and females, respectively. The individuals of* H. brasiliensis* presented total length varying from 15 to 28 cm (21.26 ± 1.89) and weight varying from 13.5 to 109 g (46.07 ± 14.54). The total length of males during the sampling period varied from 15 to 27 cm (20.82 ± 1.88) and body weight from 13.5 to 108 g (43.23 ± 14.63). The total length of females varied from 15 to 28 cm (21.52 ± 1.85) and body weight from 15.5 to 109 g (47.72 ± 14.26). The females were bigger and heavier than the males, with significant difference in total length (*t* = −3.74, df = 431, *P* < 0.05) and in body weight (*t* = −3.10, df = 431, *P* < 0.05).* H. brasiliensis* captured in the inshore waters of Caiçara do Norte, Brazil, presented similar values of total lengths registered for the same species in Venezuela [32]. The results indicate that females are bigger and heavier than males due to their gonads which tend to have higher mass compared to the testicles, thus agreeing with the results for the same species in South Florida [14, 33].

### 3.1. Sex Ratio

**Figure 2:**
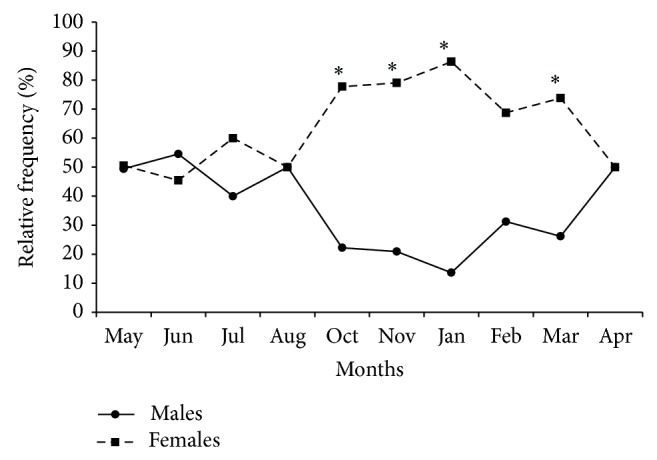
Monthly sex ratio of* H. brasiliensis*. ^∗^Significant difference of the observed sex ratio from the expected 1 : 1 (*N* males = 160; *N* females = 272).

In this study the sex ratio of* H. brasiliensis* (1 : 1.7) differed significantly (*χ* ^2^ = 29.03; *P* < 0.05) from the expected ratio (1 : 1), with a predominance of females in the sampled population. The distribution of the monthly frequency of occurrence of males and females shows significant difference in the sex ratio of* H. brasiliensis.* In the months of October, November, January, and March there was a predominance of females (Figure 2). The sex ratio could be affected by various factors related to fishery, season of the year, shoals in the feeding, and spawning areas [34–36].

Information on sex ratio is important for understanding the relationship between individuals, the environment, and the state of the population [37]. The sex ratio may vary from the expected 1 : 1 from species to species or even in the same population at different times, being influenced by several factors such as adaptation of the population, reproductive behavior, food availability, and environmental conditions [14, 38–41].

### 3.2. Length at First Sexual Maturity (*L*_50_)

**Figure 3:**
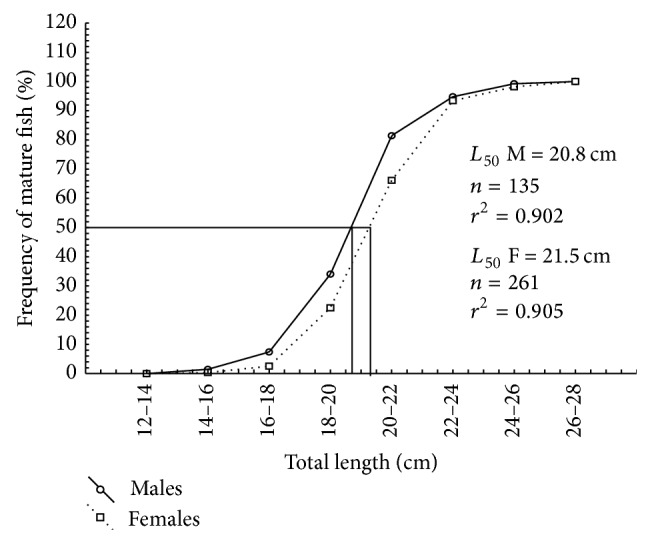
The cumulative percentage of observed mature fish in relation to body size for (a) males and (b) females of* H. brasiliensis*. The inserted lines represent the estimated size range where 50% of the fish were mature.

The total length at first sexual maturity was 20.8 cm for males and 21.5 cm for females (Figure 3). The males of* H. brasiliensis* attained first gonadal maturity at smaller body lengths than females (*t* = 3.62, df = 408, *P* < 0.05). Length at maturity could either be directly affected by changes in the quantity of energy reserves available for gonad development [42] or be indirectly affected by the changes in growth, which influence the onset of gonadal maturation [43]. The males of* H. brasiliensis* matured earlier than the females, probably because they required lesser quantity of energy reserves for gonad maturation. The females of* H. brasiliensis* in the coastal waters of South Florida attained maturity at 19.8 cm [14].

### 3.3. Macroscopic and Histological Descriptions of the Gonads

The ovaries and testes were paired bilobed structures, symmetrical, elongated, and joint in the posterior part to form a short duct leading to the urogenital pore. They were located in the posterior-dorsal part of the coelomic cavity, ventral to the kidneys and swim bladder. The immature testes were small and translucent. Maturing testes were more developed and were whitish in color. The mature testes were white and spent testes were flaccid and brown in color with hemorrhagic appearance. During maturation, the ovaries were pinkish to light orange in color and developed progressively by increasing in size and vascularization. The mature ovaries were turgid and occupied 2/3 of the coelomic cavity. The mature ovaries were turgid with numerous big oocytes visible to the naked eye, and the partially spent ovaries were flaccid. These results are similar to those registered for three fish species of the family Hemiramphidae from the coastal waters of Australia [10].

**Figure 4:**
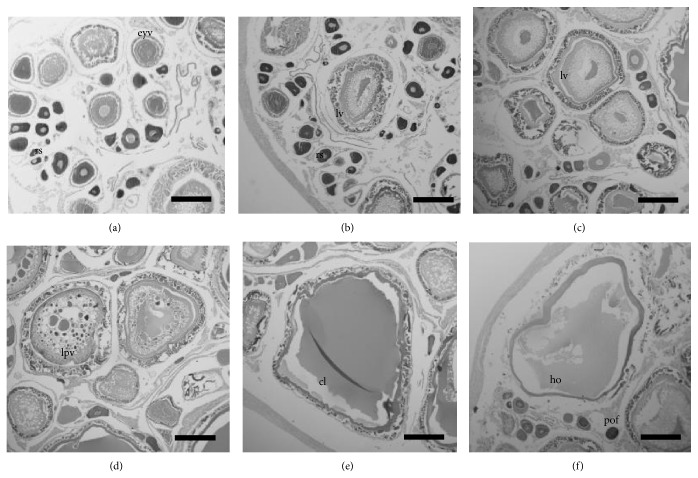
Histological aspects of oocyte development stages of* H. brasiliensis*: (a) perinucleolus stage or reserve stock (rs) and early yolk vesicle (eyv); (b) lipid vitellogenesis (lv) and rs; (c) lv (d) lipid and protein vitellogenesis (lpv); (e) oocytes with complete vitellogenesis (cl); (f) oocytes in hydration (ho) and post-ovulatory follicle (pof) (scale bar = 50 *μ*m).

Microscopic observations of the ovaries of* H. brasiliensis* showed that each lobe was a hollow sac with oocytes arranged in lamellae that extended into a central lumen. Six different phases of oocyte development were observed (Figure 4).

The pre vitellogenic phase of immature females included the young germinative cell phase of the reserve stock (Phase I). The vitellogenic stage included the beginning of lipid deposition (Phase II), lipid vitellogenic phase (Phase III) of maturing females, lipid and protein vitellogenic phase (Phase IV), complete vitellogenesis phase (Phase V), and hydrated oocyte phase (Phase VI) of mature females. Postovulatory follicles were also observed.

Phase II: initially the nucleus was in the center, with one or two nucleoli (intensely basophilic) with a well-defined cytoplasm. The nucleoli became more in number and were present in the periphery of the nucleus. Phase III (beginning of lipid deposition): this stage was characterized by small yolk vesicles in the cytoplasm. Phase IV (lipid vitellogenic phase): the oocytes showed the central nucleus, cytoplasm was less basophilic than in the previous phase and with vacuoles representing the lipid deposition. Phase V (lipid and protein vitellogenic phase): besides the lipid droplets, the oocytes showed the deposition of protein in the form of platelets in the cytoplasm. Phase VI (complete vitellogenesis phase): in this stage the lipidic droplets were not observed and the protein granules were bigger in size. The basophilic color of the cytoplasm disappeared totally and the nucleus migrated. Hydrated oocytes were highly modified; the cytoplasm was hydrated, which resulted in the adhesion of the yolk granules. Postovulatory follicles: the follicular cells were condensed, entering the space which was occupied earlier by the oocytes, thus constituting a body formed by cellular chords, folded in all directions.

The oocyte development of* H. brasiliensis* observed in the present work is in accordance with that reported from the coastal waters of southeastern Florida, USA [14].

**Figure 5:**
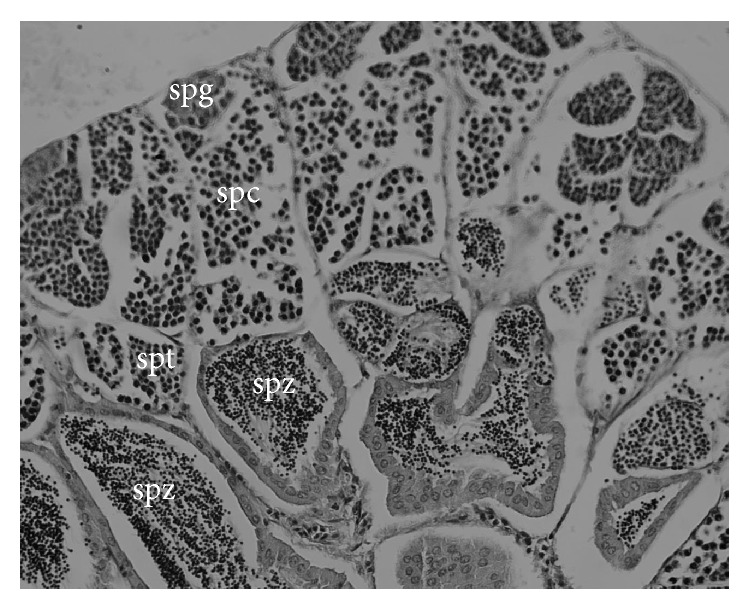
Histological aspects of spermatocyte development stages of* H. brasiliensis*: spermatocyte (spc), spermatid (spt); spermatozoon (spz) (scale bar = 50 *μ*m).

Histological analyses indicated four developmental phases of the spermatogonia in* H. brasiliensis*: spermatogonia, spermatocytes, spermatids, and spermatozoa (Figure 5).

In an earlier study of the ballyhoo,* H. brasiliensis*, it was observed that the testis type was restricted lobular, where spermatogonia were restricted to the distal termini of lobules rather than being distributed along the lobules [44]. In the present study, it was observed that the testicles of maturing males had spermatogonia and developing germinative cells. In mature males the testis lobules were full of spermatogonia and there was a reduction in the diameter size of tubules of spent fish with residual spermatogonia. The development was consistent along the whole length of the testis depending on the degree of maturation.

### 3.4. Type of Spawning and Fecundity

**Figure 6:**
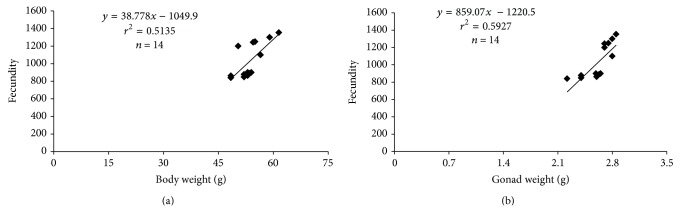
(a) Relationship between fecundity and body weight of mature females (g); (b) relationship between fecundity and gonad weight (g).

The microscopic characteristics of gonad development of* H. brasiliensis* indicated multiple spawning. The batch fecundity of* H. brasiliensis* varied from 862 to 1354, with an average of 1153 (±258.22) vitellogenic oocytes for 50 g body weight of female.  Figure 6 shows the relationship between fecundity and body weight of mature females and between fecundity and gonad weight (g). Fecundity increased with bigger body size and also with increasing gonad weight. For the same species in the coastal waters of south of Florida, the fecundity was 1164 oocytes for 100 g body weight of female [14]. Fecundity is a specific reproductive tactic [23] and is adapted to the life cycle conditions of the species [45], varying with growth, population density, body size, food availability, and mortality rate [33]. The life-history traits that typically represent trade-offs in evolutionary terms are balanced, so that the lifetime egg production of* H. brasiliensis* is the same order of magnitude in the present study and as well in an earlier study for the same species in the coastal waters of south Florida [14].

### 3.5. GSI, Reproductive Period

**Figure 7:**
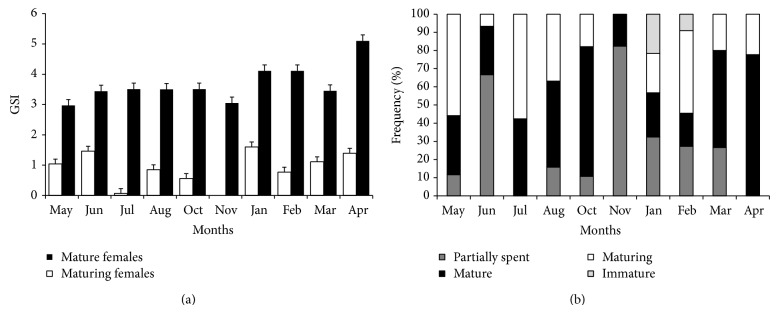
(a) Temporal dynamics of GSI (±SE) of* H. brasiliensis* maturing and mature females; (b) monthly frequency of maturity stages of the females of the* H. brasiliensis* (*N* females = 272).

The mean monthly values of GSI of maturing and mature females are shown in Figure 7(a). The GSI of mature females presents the highest value during the month of April. Frequency of monthly gonadal maturation stages of females indicates that mature females occur throughout the year (Figure 7(b)).* H. brasiliensis* has a prolonged reproductive period, almost throughout the year, with a peak in April. Reproductive biology of* H. brasiliensis* and* H. balao* relating to maturation, spawning frequency, and fecundity were reported from the coastal waters of southeastern Florida [14], when it was observed that breeding occurs in the month of April with peaks during the end of spring and the beginning of summer. The results of the present paper are similar to the earlier findings [14], since the highest GSI of mature females was in April.

This study indicates that females predominate in the sampled population of* H. brasiliensis.* The females were larger in size than the males; however, the males attained sexual maturity earlier than the females. The macroscopic gonadal stages coupled with histological analysis reflect the spawning activities of* H. brasiliensis* adequately. A study on otoliths indicated that* H. brasiliensis* lives for four years in the coastal waters of southeastern Florida, USA [14]. Based on the age and reproductive aspects of this species (small size, early maturity, and short life span) it is considered as an opportunistic strategist. This study provides information on the reproductive aspects, such as sex ratio, length at first sexual maturity, gonad development, and fecundity besides the reproductive strategy of* H. brasiliensis* from the coastal waters of Northeastern Brazil.

## 4. Conclusion

This study confirms that the females of* H. brasiliensis* are larger and heavier than males, which mature before them. The macroscopic characteristics of the gonads indicate four maturation stages. Histological studies of gonads showed six phases of oocyte development and four phases of spermatocyte development.* H. brasiliensis* is a multiple spawner, with a prolonged reproductive period during the whole year, presenting a peak in the month of April, and is considered as an opportunistic strategist.
